# Interactions between Nanoclay, CTAB and Linear/Star Shaped Polymers

**DOI:** 10.3390/ijms23063051

**Published:** 2022-03-11

**Authors:** Elżbieta Grządka, Ewelina Godek, Grzegorz Słowik, Agnieszka Kowalczuk, Jakub Matusiak, Urszula Maciołek

**Affiliations:** 1 Department of Radiochemistry and Environmental Chemistry, Institute of Chemical Sciences, Faculty of Chemistry, Maria Curie-Skłodowska University, M. Skłodowskiej-Curie 3 Sq., 20-031 Lublin, Poland; ewelina.godek@poczta.umcs.lublin.pl; 2 Department of Chemical Technology, Institute of Chemical Sciences, Faculty of Chemistry, Maria Curie-Skłodowska University, M. Skłodowskiej-Curie 3 Sq., 20-031 Lublin, Poland; grzegorz.slowik@mail.umcs.pl; 3 Centre of Polymer and Carbon Materials, Polish Academy of Sciences, M. Curie-Skłodowskiej 34, 41-819 Zabrze, Poland; akowalczuk@cmpw-pan.edu.pl; 4 Faculty of Chemistry, Wroclaw University of Science and Technology, Wybrzeże Wyspiańskiego 27, 50-370 Wroclaw, Poland; jakub.matusiak@pwr.edu.pl or; 5 Analytical Laboratory, Institute of Chemical Sciences, Faculty of Chemistry, Maria Curie-Skłodowska University, M. Skłodowskiej-Curie 3 Sq., 20-031 Lublin, Poland; urszula.maciolek@poczta.umcs.lublin.pl

**Keywords:** stability, adsorption, nanoclay, polyacrylic acid, CTAB

## Abstract

The influence of star-shaped (PAA-SS) and linear polyacrylic acid (PAA) with different molecular weights (high—PAA-HMW and low—PAA-LMW) on the structure of the adsorption layer, adsorption amount, electrokinetic and stabilizing properties of the PAA/CTAB/nanoclay suspensions was studied. The properties of the systems containing one of these polymers, the cationic surfactant—hexadecyltrimethylammonium bromide (CTAB) and the surface-modified nanoclay (N-SM) were analyzed using the following techniques: BET, CHN, FT-IR, ED-XRF, XRD, HRTEM, UV-Vis, tensiometry and zeta potential measurements. It was proved that PAA could be used as an effective stabilizer of N-SM. Moreover, the addition of CTAB caused a significant increase in the stability of the systems but decreased the adsorption of PAA on the N-SM surface and changed the structure of the adsorption layers. The largest stability was observed in the PAA-HMW/CTAB system. The PAA polymers and PAA/CTAB complexes adsorbed, especially on the clay surface, influenced the primary distribution of the layered sheets but kept the same basal d-spacing. The adsorption of PAA and the PAA/CTAB complexes took place mainly at the plate edges and on the contact space between the sheets. The obtained results will be used for the preparation of the PAA/CTAB/nanoclay composite for water purification.

## 1. Introduction

The contamination of water has become a critical problem due to potential health concerns [1]. Many processes can be used for the removal of pollutants from water. Adsorption is one of them, and a large number of novel adsorbents, such as polymer/nanoclay composites (PNC), have been developed in recent years. PNC are characterized by large surface area, high adsorption capacity and an excellent life cycle for water treatment, effectiveness and remediation potential, as well as their relatively low cost and toxicity [2]. It should be emphasized that PNC are also used in many industries where conventional, monolithic materials do not show satisfactory properties or are characterized by too high own weight. However, before the successful preparation of such materials, preliminary research concerning the interactions between potential components of PNC should be conducted.

Nanoclays are natural nanoparticles of nanometre thick two-dimensional silicate layers arranged together. They exhibit a chemical structure similar to montmorillonite. Unlike conventional clays, nanoclays are clay minerals that have at least one dimension in the nanometer range. Nanoclays dispersed homogeneously in the hydrogel network improve the thermal, mechanical and barrier properties of the polymer systems significantly [3,4,5]. The replacement of the natural inorganic cations in the clays with other organic ones has been extensively studied in order to change the surface properties of the clays and improve their adsorption capacity. These ions reduce the surface energy of the clay, making it more organophilic, thus facilitating access between the layers [6]. The modification process can cause a significant change in the surface properties as well as affect the pore structure of the clays [5]. The nanoclays are also known as are non-toxic and environmentally friendly. These substances have been used as excellent adsorbents and catalysts [6,7,8,9].

The introduction of mineral nanofillers such as clays into polymers matrix composites is known as one of the techniques allowing to improve the properties of the hybrid materials and to expand their application fields. However, to change their hydrophilic character, lamellar clays must be organically modified to increase their dispersion in the hydrophobic polymer matrix. PNCs may be fabricated using various techniques, such as in situ synthesis, solution mixing, melt blending, electrospinning, etc. Several attempts to prepare PAA/clay-based composites by employing various techniques have been reported [10,11,12,13]. The selection of the preparation mode depends on various parameters, such as the used polymeric system, the application field, particle size distribution, etc. Control of the colloidal properties and stability of the concentrated clay dispersions is of great importance in the manufacturing of high-quality products such as paper coatings, cosmetics and paints. Although layered clay particles can be well dispersed and swell in water, the addition of intercalating agents is necessary to obtain a fully exfoliated clay dispersion. Together with poly(sodium acrylate), poly(acrylic acid) PAA is one of the most commonly used water-soluble anionic polyelectrolytes. Such properties as hydrophilicity, nontoxicity and binding capacity are valuable in the production of hydrogels, superabsorbents, ion exchange resins and in dispersing and binding agents [14,15,16,17,18]. Furthermore, due to their low toxicity, they are used as food additives [19].

Adsorption is considered to be the most convenient process among the various methods of water treatment and is commonly used to remove low concentrations of non-degradable organic compounds from wastewater and industrial effluents. Many papers have reported the use of PAA-based composites fabricated by various techniques to remove dye such as methylene blue [10,20,21,22] and safranine T [23]; metal ions like zinc, nickel, magnesium, copper and lead [24]. Recently, bio-based composites have been subject to increased interest due to their non-toxic properties, biodegradability and surface reactivity. Very few works have demonstrated the developed biocomposites with modified kaolinite-rich clay using chitosan and cellulose nanocrystals, evaluating their adsorption capacity by studying adsorption for chromium(VI) and methylene blue solution [25,26,27].

Since water purification is of great importance to society, the influence of surfactants on the stability of the colloidal suspensions should be examined. It was previously confirmed [28] that the small addition of surfactants lowered the efficiency of the used flocculant. In the current studies, the influence of cationic surfactant CTAB on the stability of aqueous suspensions of modified nanoclay was evaluated. Due to the possible applicability of the studied materials in the process of water purification, the obtained results, once again, show that it is extremely important to study how each of the components affects the system’s stability. As one can see, stability and the adsorption properties of the polyacrylic acid/N-SM suspensions in the presence and absence of surfactants have never been investigated so far. The other novelty of the presented results is the usage of the star polymers possessing completely different properties compared to the linear ones. Such substances have never been adsorbed onto the surface of clay minerals. However, their adsorption on the silica/aqueous interface was analyzed [29]. The authors believe that the use of the star-shaped polymers as adsorbates on the surface of clay minerals could extend not only scientific knowledge with very interesting results but also find unique applications, especially in the preparation of novel PNCs. The star-shaped polymers belong to the group of branched polymers. They are made of a central structural element—the core, covalently bound to the arms—chains of linear homo- or copolymers [30]. For many years studies in the field of polymeric stars have focused on their structure control. However, recently due to the relative ease of synthesis, possible high molar masses, multifunctionality and therefore unique intrinsic properties, they are of significant interest for various applications, mainly in the host-guest encapsulation and drugs delivery in medicine [31] as well as compatibilizers [32], emulsifying agents [33] or viscosity improving additives [34]. The complex architecture of the star structures influences their physical properties significantly. The differences in the properties of the stars compared to their linear analogues can be seen in both solutions and the solid state. The higher segment density of the branched polymers compared to those of linear structure determines their solution properties to a great extent. The dimensions (hydrodynamic volumes) occupied by such macromolecules in solutions are smaller in comparison with the linear chains of the same molar mass. The compact shape of the branched structures and the lack of entanglements between the macromolecules decide about their better solubility in comparison to their linear analogues [35]. The comparisons between the linear polymers and the star polymers have been the subject of investigations in many reports [36,37]. For example, the properties of the stars with polyacrylamide arms such as salt resistance, alkali resistance and compatibility with surfactants were found to be much better than those of linear polyacrylamide [36].

The aim of this study was to study the structure and properties of the polyacrylic acid/CTAB/nanoclay adsorption layer in the systems with different PAAs (linear and star-shaped) and different chain lengths (M_w_ = 100,000 Da and M_w_ = 1,300,000 Da) using the following techniques: BET, HRTEM, CHN, FT-IR, ED-XRF, XRD, UV-Vis spectrophotometry, tensiometry and zeta potential measurements. Two hypotheses that were checked are: (i) small amounts of CTAB in the PAA/N-SM system will completely change the stabilizing and adsorptive properties of the system as well as the structure of the formed adsorption layer and (ii) the molecular weight of PAA has a larger influence on the stabilizing and adsorptive properties that the structure of used PAA. The results of such studies are very promising and provide great application potential in many branches of industry, especially in water treatment. Presented studies are preliminary research concerning the interactions between the potential components of PNCs. Knowledge about their interactions will enable obtaining the optimal composite characteristics. The obtained results are crucial for the preparation of the polyacrylic acid/CTAB/nanoclay composite for water purification.

The specific objectives of this work were:

- studying the interactions between CTAB and PAA of different molecular weights and structures
- determination of the influence of the PAA structure, its molecular weight and the presence of CTAB on the stabilizing and adsorptive properties of the PAA/N-SM system
- studying the structure of the adsorption layer in the PAA/N-SM and PAA/CTAB/N-SM systems, determination of the shapes of the formed lamellae and precise indication where exactly the adsorption process takes place
- an indication of the best conditions to obtain the most stable PAA/N-SM system.

## 2. Results and Discussion

### 2.1. Adsorption Measurements

**Figure 1:**
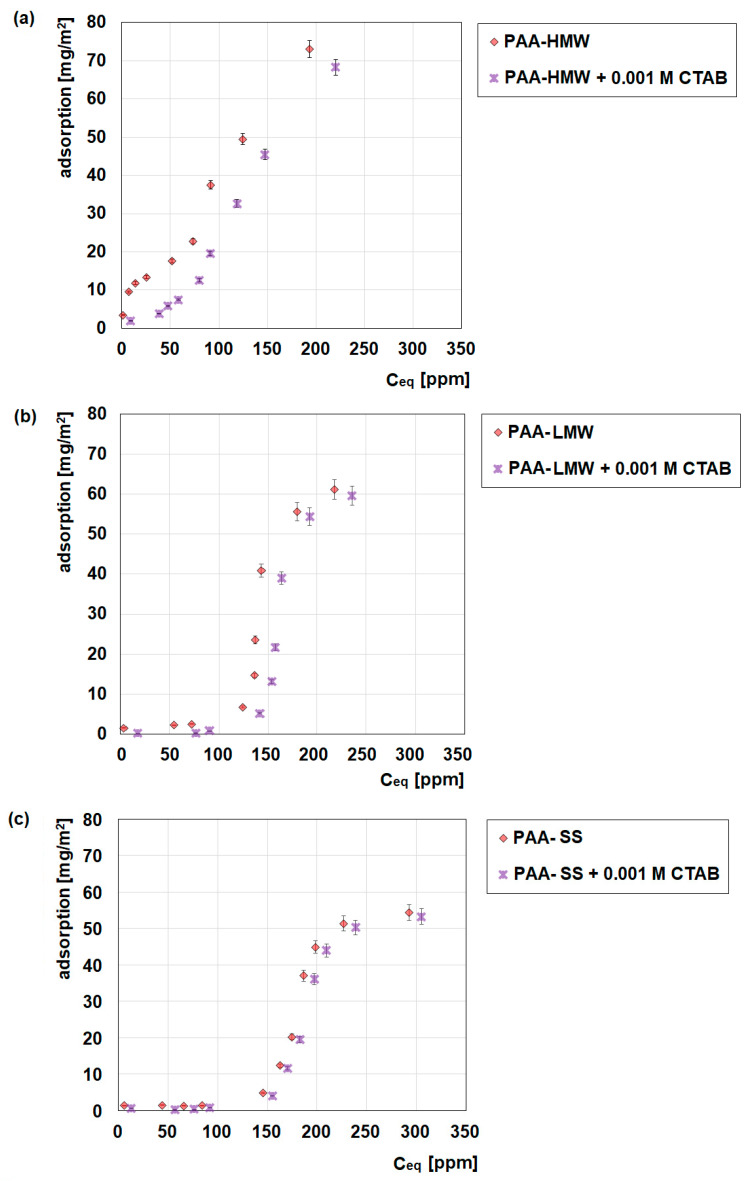
Influence of CTAB (0.001 mol/dm^3^) on the adsorption of polyacrilic acid on the nanoclay surface, 0.01 mol/dm^3^ NaCl, pH = 7, (**a**) PAA-HMW, (**b**) PAA-LMW, (**c**) PAA-SS.

UV-Vis spectrophotometry allows for an estimation of the amount of PAA or PAA/CTAB complexes on the N-SM surface, which is helpful for the preparation of composites. Figure 1 shows the influence of CTAB on the amount of polyacrylic acid adsorption on the nanoclay surface. The obtained data prove that polyacrylic acids easily adsorb on the nanoclay surface. Due to the anionic nature of the polymers and the existence of positively charged groups on the nanoclay surface (trimethyl stearyl ammonium chains), it can be concluded that the electrostatic interactions can be responsible for the adsorption process [38]. However, other types of interactions, for example, hydrophobic or hydrogen bonding, are also possible in this system. Moreover, S-shaped isotherms are obtained in the analyzed systems [39]. Such a shape of isotherm is the consequence of two effects. First, the attractive forces between the solutes at the surface may cause cooperative adsorption. Second, the sorption of a solute may be inhibited by a competing reaction within the solution, such as a complexation reaction with a ligand [40]. These findings are in agreement with Limousin’s statement that S-shaped isotherms are always the result of at least two opposite mechanisms [41]. An example is the cooperative adsorption between the non-polar organic compounds and the surfactants on the clay surface. Such non-polar substances possess a small affinity for the surface of the clay, but as soon as the clay surface is covered by some molecules, other organic molecules are adsorbed more readily and effectively [41]. As far as the influence of the polymer molecular weight is concerned, it can be seen that if the molecular weight increases, the amount of the polymer adsorption is also larger (Figure 1a,b). The explanation is connected with the fact that if the number of the adsorption sites on the surface of the adsorbent is constant, the adsorption of the larger polymer of higher molecular weight causes the increase in the total amount of the polymer adsorption. The obtained data also allow the drawing of some conclusions about the influence of the polymer structure on the adsorption process. As one can see, the adsorption amount of the star-shaped PAA is the smallest among the three measured systems. The explanation can be found in the structure of the star-shaped PAA, which on average contains ten arms in its macromolecule, each of which is close to 10,000 Da in length. Due to its topology and probably steric reasons, this type of polymer is less adsorbed on the nanoclay surface. However, it should be stressed that the differences between PAA-SS and PAA-LMW of comparable values of the molecular weight are not large; however, they are noticeable.

**Figure 2:**
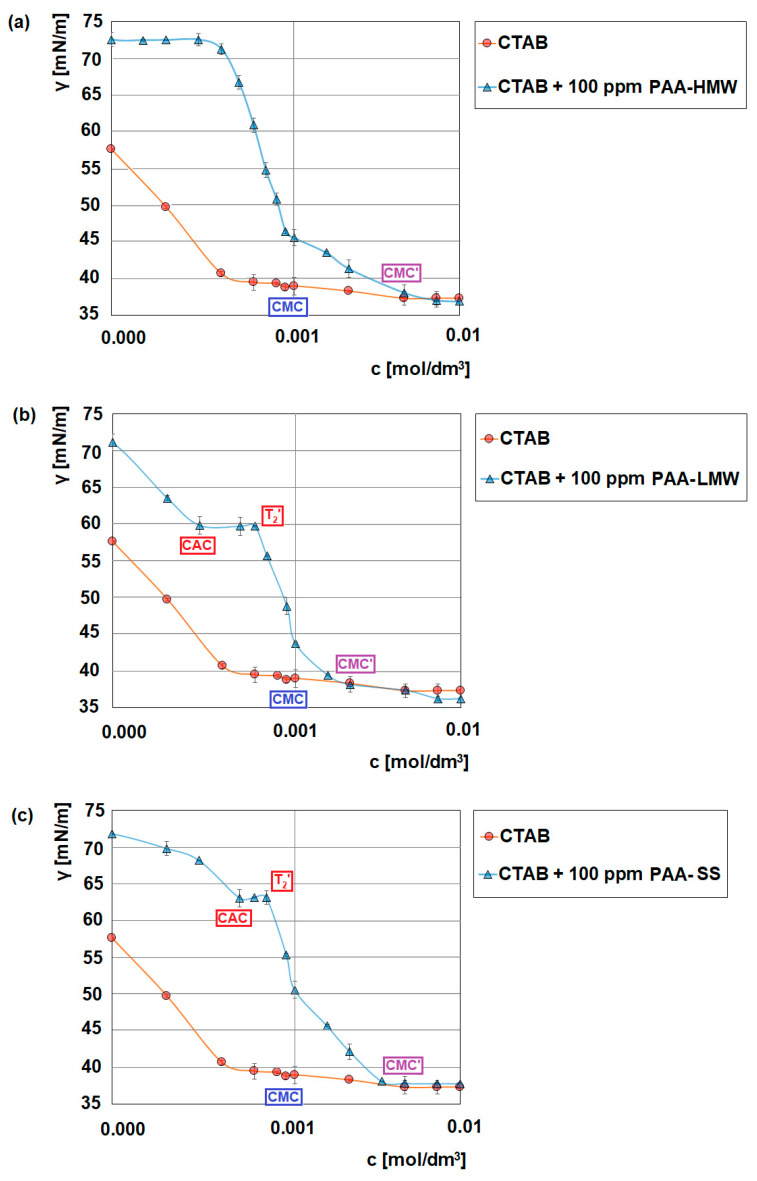
Influence of polyacrylic acid (100 ppm) on the surface tension of CTAB, (**a**) PAA-HMW, (**b**) PAA-LMW, (**c**) PAA-SS.

**Figure A1:**
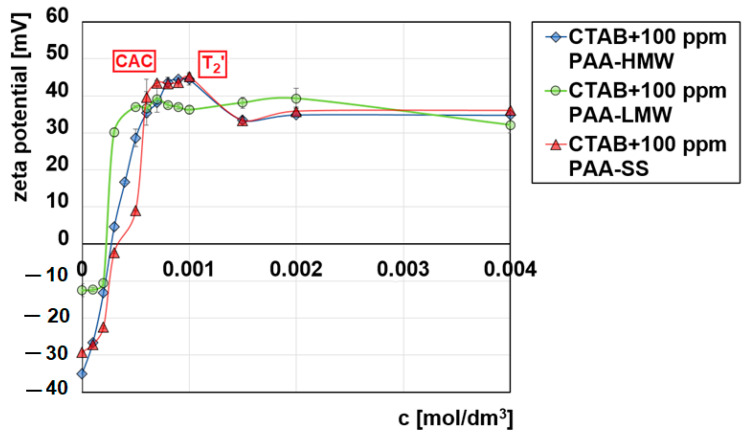
Influence of PAA (100 ppm) on the zeta potential of CTAB.

The addition of CTAB to the adsorption systems causes a small decrease in the PAA adsorption amount. The formation of the polymer-surfactant complexes with a small affinity for the adsorbent surface might be responsible for this situation (Figure 2). If the interactions between the formed complexes and the adsorbent surface are weaker in comparison to those between the PAA macromolecules and the nanoclay surface, the deterioration of the PAA adsorption is observed [42]. There is also another explanation. If the mechanism of PAA macromolecules adsorption on the nanoclay surface is mostly the electrostatic attraction coming from the interactions between the anionic macromolecules and the positively charged nanoclay surface, the addition of cationic CTAB, which can form the polymer-surfactant complexes of the positive charge (Figure A1) with PAA macromolecules, can cause the deterioration of the adsorptive properties in the system [43,44].

### 2.2. Polymer-Surfactant Complexes in Solutions

Figure 2 shows the influence of different structures and molecular weights of the polyacrylic acid samples on the surface tension of CTAB. The surface tension allows us to gain some information about the formation of the PAA/CTAB complexes. These measurements also show under which conditions the above-mentioned complexes are created. These complexes have a significant influence on adsorption, stability and the structure of the adsorption layers. The first observation from the obtained data is that the value of the lowest surfactant concentration at which micelles begin to form—the critical micellization concentration (CMC) is around 0.0009 mol/dm^3^, which is consistent with the literature data [45]. The addition of the macromolecular compound to the surfactant solution causes strong interactions between the oppositely charged polymer chains and the surfactant molecules. The effect is the formation of the polymer-surfactant complexes [46]. Several types of interactions between the surfactant and the oppositely charged polymer can be distinguished [47]. One of them is the associative interactions resulting from the electrostatic attraction, as a result of which the charge is neutralized. Another type is the hydrophobic interaction as a result of the interactions causing the aggregates composed of the surfactant molecules to form along the polymer chain [47,48]. On the surface tension curve of the surfactant in the presence of the polymer, a few characteristic points occur. Except for the above-mentioned CMC, the CAC, T_2_′ and CMC’ points can be distinguished. CAC is a critical aggregation concentration. This is the smallest concentration when the surfactant-polymer interactions start. The CAC is usually much lower than the CMC of the surfactant when the electrostatic interactions between an ionic polymer and an oppositely charged ionic surfactant are involved. However, if the hydrophobic interactions occur in the measured systems, the CAC is closer to the CMC point. It was possible to estimate the CACs values for the CTAB/PAA-LMW (0.0003 mol/dm^3^) and for the CTAB/PAA-SS (0.0005 mol/dm^3^) systems. However, in the case of the CTAB/PAA-HMW system, it was impossible to estimate the value of the critical aggregation concentration. Probably this fact results from the strong polymer-surfactant interactions starting at a very small surfactant concentration [49]. The other mentioned critical surfactant concentration—T_2_′ refers to a surfactant concentration where the polymer chains become saturated with the bound surfactant molecules or micelles [50,51]. T_2_′ for the CTAB/PAA-LMW system is equal to 0.0006 mol/dm^3,^ whereas its value for the CTAB/PAA-SS system is 0.0007 mol/dm^3^. It should also be emphasized that in the case of weak interactions between the surfactants molecules and the polymer chains, CAC and T_2_′ values are usually close to that of CMC of the pure surfactant [50]. When taking into consideration the obtained values of the critical points in the studied systems, it can be concluded that the interactions between the CTAB molecules and the PAA chains of different length structures are strong. The formation of the polymer-surfactant complexes between the cationic surfactant and the anionic polymer proceeds very effectively in the area between the CAC and T_2_′ points. In order to analyze the formed complexes in detail, it is necessary to refer the obtained results of the surface tension measurements to the zeta potential ones (Figure A1). The zeta potential measurements provide important information about the charge of the above-mentioned complexes, which is crucial in terms of the adsorption mechanism. As can be seen, the zeta potential values for the pure polyacrylic acid solutions are approximately: −35 mV (for polyacrylic acid with a high molecular weight), −12 mV (for polyacrylic acid with a low molecular weight) and −29 mV (for star-shared polyacrylic acid). The results show that at a low CTAB concentration, as the surfactant concentration increases, a gradual charge neutralization and the increase in the zeta potential values can be observed. This is evidence of the electrostatic polymer—surfactant complex formation. Whereas at higher CTAB concentrations, when the values of the zeta potential in the systems are almost constant (the CAC- T_2_′ range), the non-electrostatic mechanisms of the polymer-surfactant interactions (hydrogen bonding, hydrophobic ones) are very probable. As follows in the studied systems, both the electrostatic and non-electrostatic interactions can be observed. Above the concentration corresponding to the T_2_′ point, the zeta potential remains constant, which indicates that the PAA chains are fully saturated with the CTAB molecules. Another important observation is that the zeta potential of the formed complexes is still positive, which also means that besides the electrostatic interactions between the surfactant molecules and the PAA chains, other types of interactions, for example, hydrogen bonding or the hydrophobic ones, can also occur [52].

### 2.3. HRTEM

**Figure 3:**
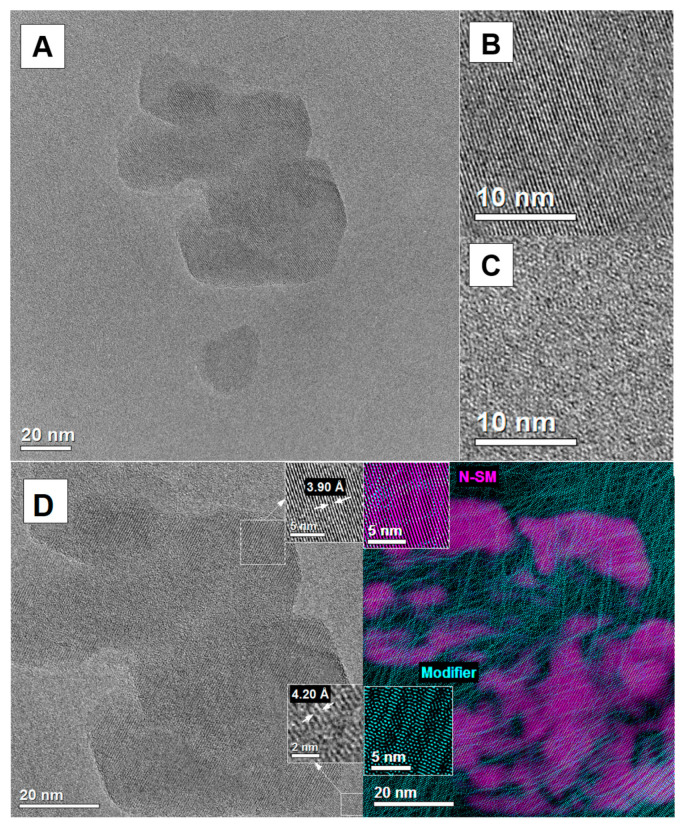
TEM and HRTEM images of the N-SM sample: TEM image of N-SM (**A**), HRTEM image of N-SM (**B**), HRTEM image of modifier (**C**), HRTEM images of the N-SM sample with the phase identification (**D**).

**Figure 4:**
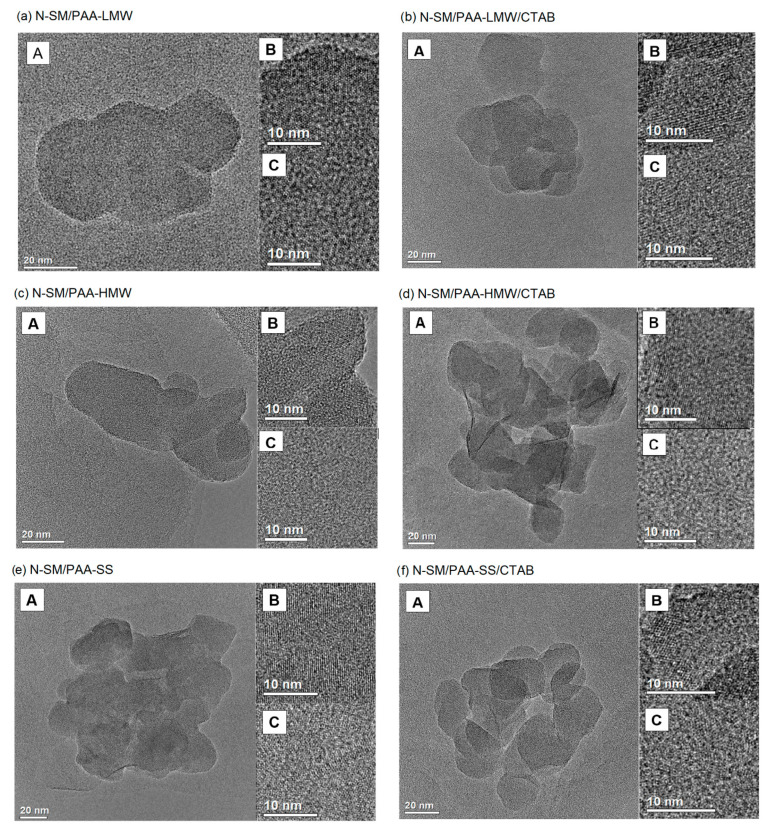
TEM images of the studied samples (**A**), HRTEM image of N-SM (**B**), HRTEM image of PAA or PAA/CTAB mixture (**C**).

**Figure 5:**
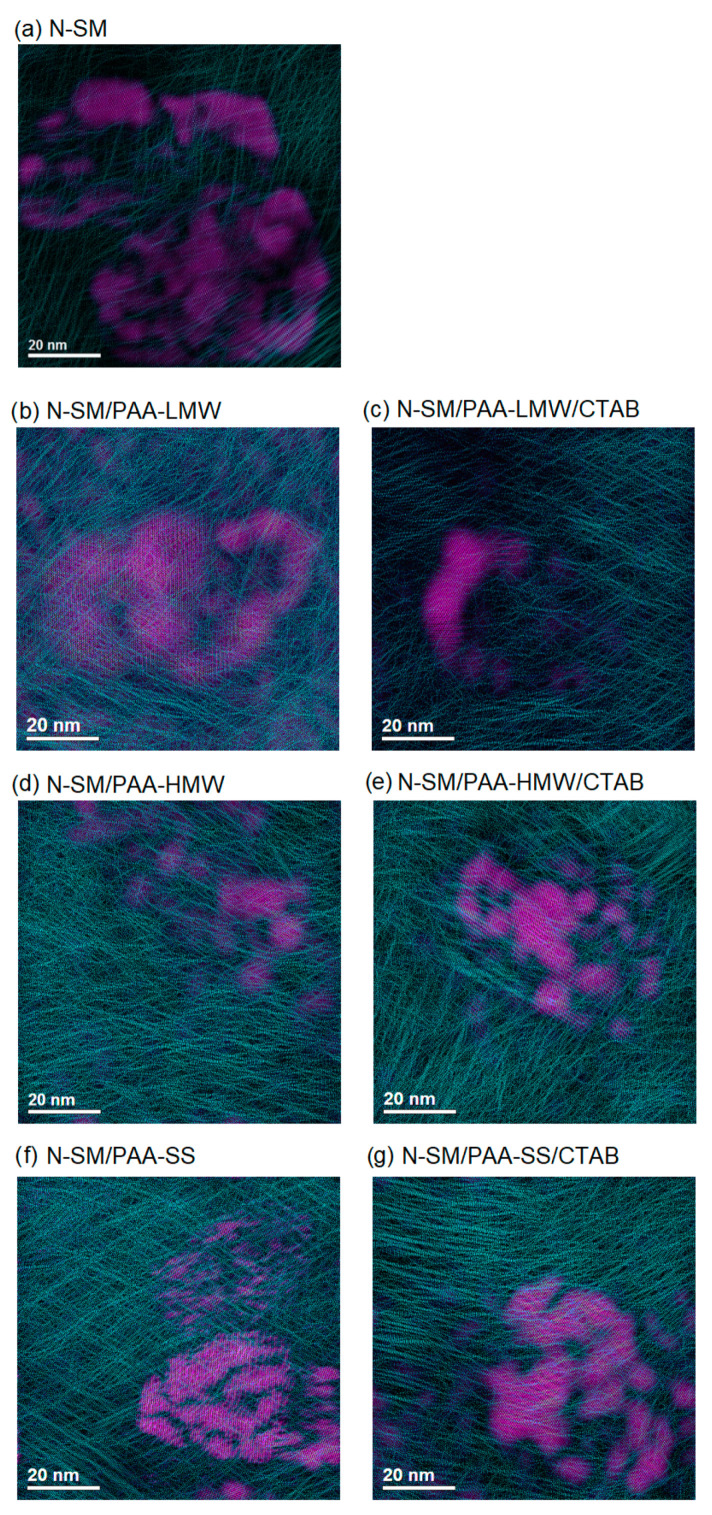
Distribution of chains in the polymer matrix before (**a**) and after adsorption of PAA or PAA/CTAB complexes on the N-SM surface (**b**–**g**). The purple color indicates areas of the sample that are poorly covered with the polymer layer.

HRTEM provides information about the shapes of the formed lamellae and helps to indicate where exactly the adsorption process takes place. Moreover, this method shows where the intercalation occurs, which is extremely important in the preparation of composites. Figure 3, Figure 4 and Figure 5 show the HRTEM images of the samples. Before imaging, the samples were dispersed (sonicated) in ethyl alcohol, then transferred to microscope grids and dried. All obtained preparations (particles) consist of few phyllosilicate layers and are much thinner in depth than they are in the lateral dimension. Depending on the sample, their morphologies are different. The N-SM sample (Figure 3A) exhibits a spherical lamellar morphology. The lamellas in the aggregate are not uniformly covered with the modifier, and disordered chains can be observed (Figure 3C). The lattice planes of lamellas are well visible in the HRTEM images (Figure 3B). The distances between the atoms in the N-SM lamella observed in the HRTEM images (Figure 3D) are 3.90 Å. In contrast, the distance between atoms of the chains in adjacent chains of the TMSA modifier is 4.20 Å. The distribution of the modifier on the surface of the lamella is marked in green. The areas with a dominant crystal structure are colored purple. Figure 3D shows that the N-SM surface is unevenly covered with the chains of the TMSA expander.

TEM and HRTEM images of functionalized samples are shown in Figure 4a–f. The phyllosilicate lamellas in the sample N-SM/PAA-LMW are characterized by the plate-like morphology, and the particles of N-SM are in the form of aggregates embedded in the PAA-LWM mixture (Figure 4a). The polymer covers the lamellas quite evenly, so their crystal structure is completely invisible, and a thick layer of the polymer is accumulated at the edges of the lamella (see insert B). The grains of the N-SM/PAA-LMW/CTAB sample are compact aggregates (Figure 4b). The lamellas in the sample assume a circular morphology and overlap. Furthermore, the N-SM particles are arranged in the layers and stuck with the polymer/surfactant mixture. In the N-SM/PAA-HMW, sample the phyllo-silicate sheets are compact and exhibit a circular morphology (Figure 4c). The adsorbed polymer is characterized by the flat chains conformation and covers the N-SM surface unevenly. A much thicker layer of PAA-HWM can again be observed at the lamellas envelope (see insert B). The N-SM/PAA-HMW/CTAB sample is characterized by loosely arranged platelets with a circular morphology (Figure 4d). When CTAB is added into the sample, the platelet reorganization occurs, and the formed aggregates are larger. A much thicker layer of the polymer can be observed at the lamella edges (see insert B). In addition, a few exfoliated sheets stacked perpendicularly to the plane can also be observed. However, this effect could also be due to the sonication of the sample prior to TEM imaging. The particles in the N-SM/PAA-SS sample show spherical morphology (Figure 4e). The lamellas are rounded and rather poorly covered with the polymer whose twisted chains are clearly visible in the B panel. The thin black lines in the TEM image represent some exfoliated lamellas. This indicates that some macromolecules of PAA-SS are pressed between the adsorbent sheets making its structure quite loose. It results from the star-like structure of the polymer, which allows for the penetration of the interlayer gaps and causes their intercalation. In the N-SM/PAA-SS/CTAB sample, much larger distances between the lamellas can be observed (Figure 4f). The particles show a spherical morphology and appear in the form of rather loosely packed plates. The addition of the CTAB to the sample increased the distance between them due to the existence of the PAA-SS/CTAB complexes. The phyllosilicate sheets in the sample are nearly completely covered with the polymer, and the N-SM crystal structure is barely visible (see panel B).

HRTEM images (Figure 5) show a progressive increase in the chain packing density (turquoise color) on the N-SM clay surface (purple color) and in its surrounding after the PAA and CTAB adsorption. In addition, the chain arrangement following the adsorption of the PAA/CTAB complex has changed. After this process, it is oriented and organized less randomly changed. Moreover, a significant concentration of the polymer long chains at the edges of the sample is observed.

**Figure A2:**
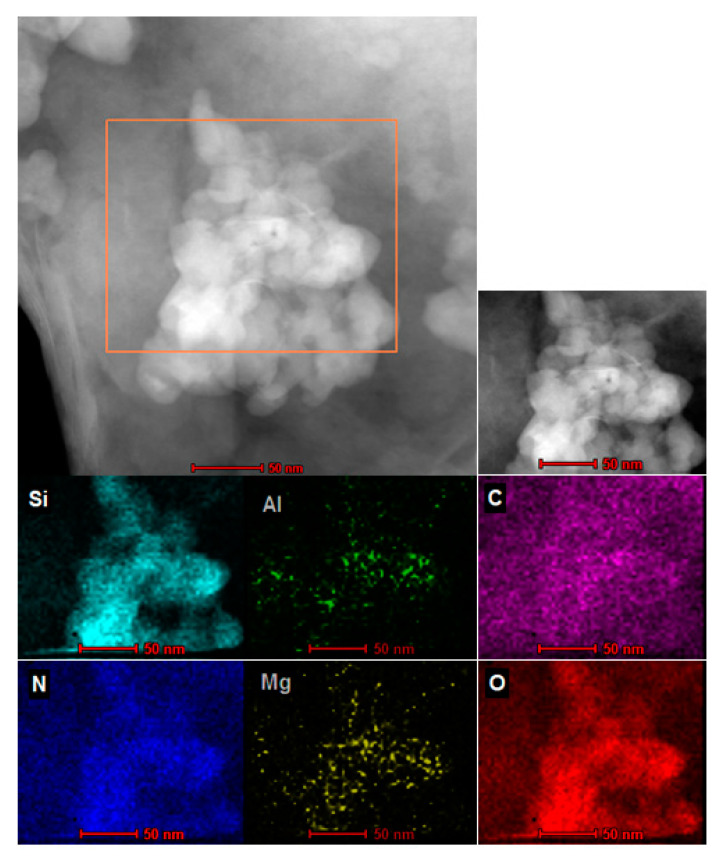
STEM-EDS analysis of the N-SM sample. Top: STEM image of the analyzed object (MMT particle composed of aggregated lamellae); bottom: chemical mapping showing the distribution of the atoms within the boxed area.

**Figure A3:**
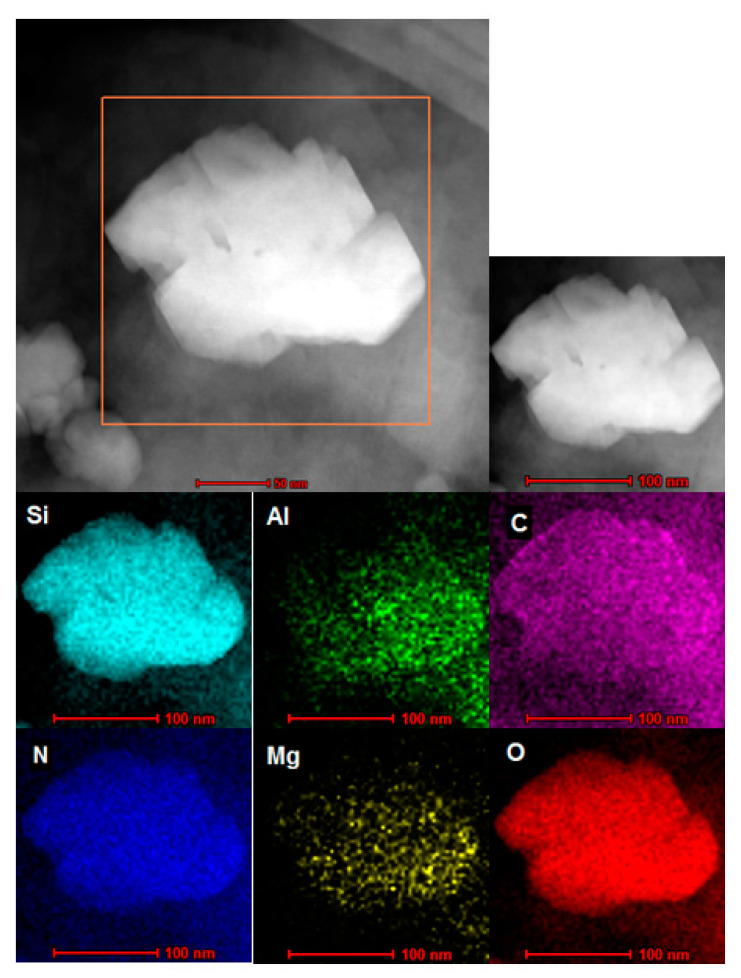
STEM EDS analysis of the N-SM/PAA-LMW sample. Top: STEM image of the analyzed object (MMT particle composed of aggregated lamellae); bottom: chemical mapping showing the distribution of the atoms within the boxed area.

**Figure A4:**
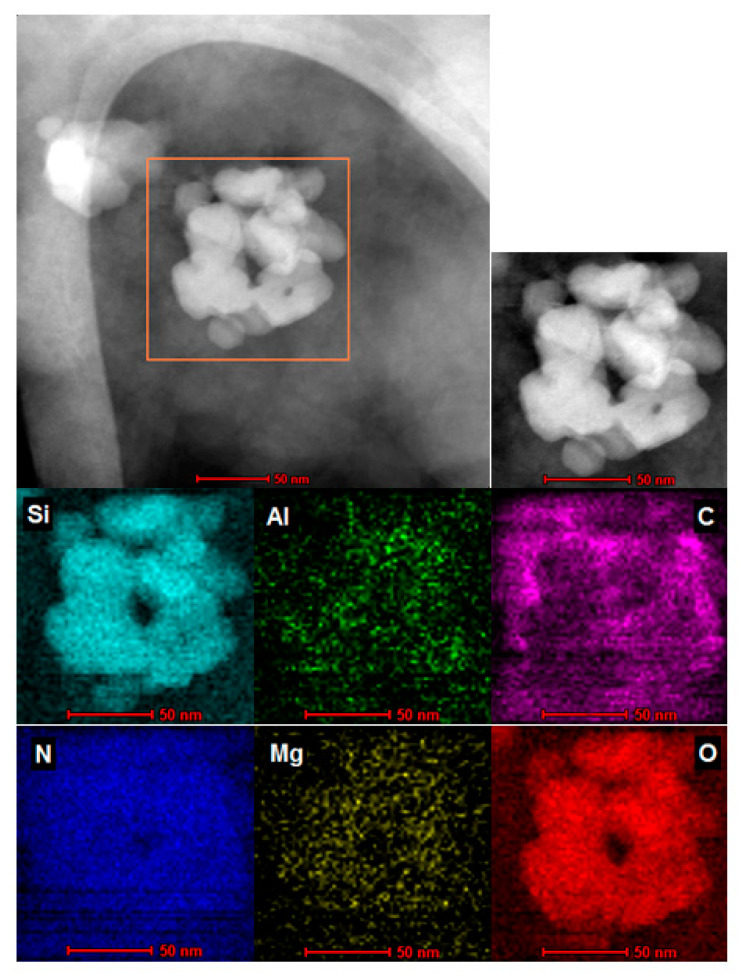
STEM EDS analysis of the N-SM/PAA-SS sample. Top: STEM image of the analyzed object; bottom: chemical mapping showing the distribution of the atoms within the boxed area.

**Figure A5:**
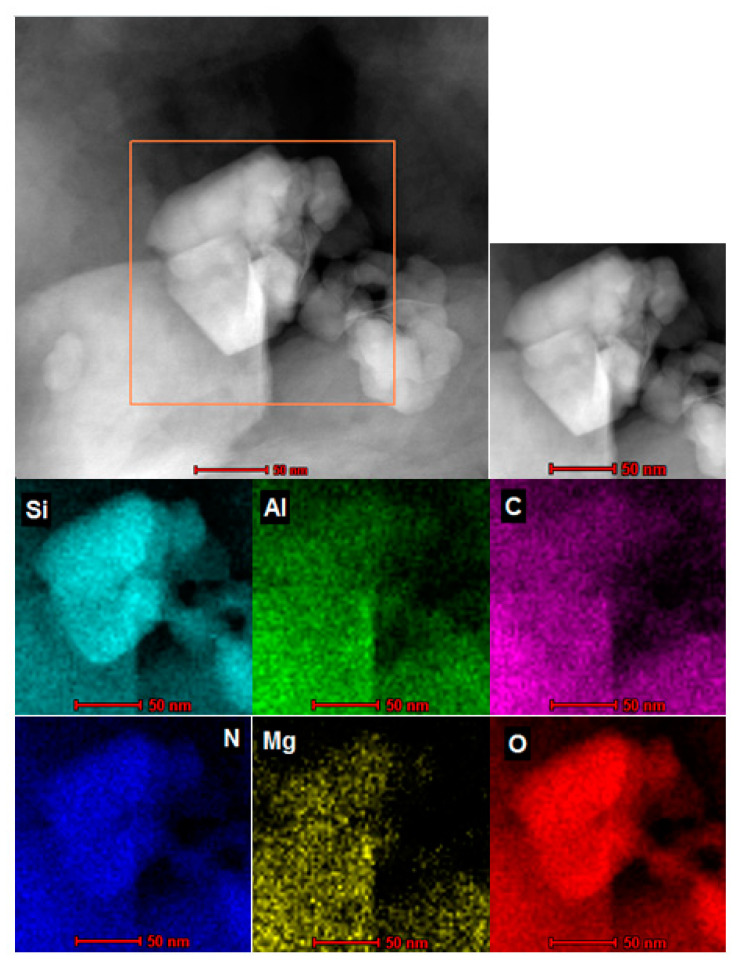
STEM EDS analysis of the N-SM/PAA-LMW/CTAB sample. Top: STEM image of the analyzed object; bottom: chemical mapping showing the distribution of the atoms within the boxed area.

**Figure A6:**
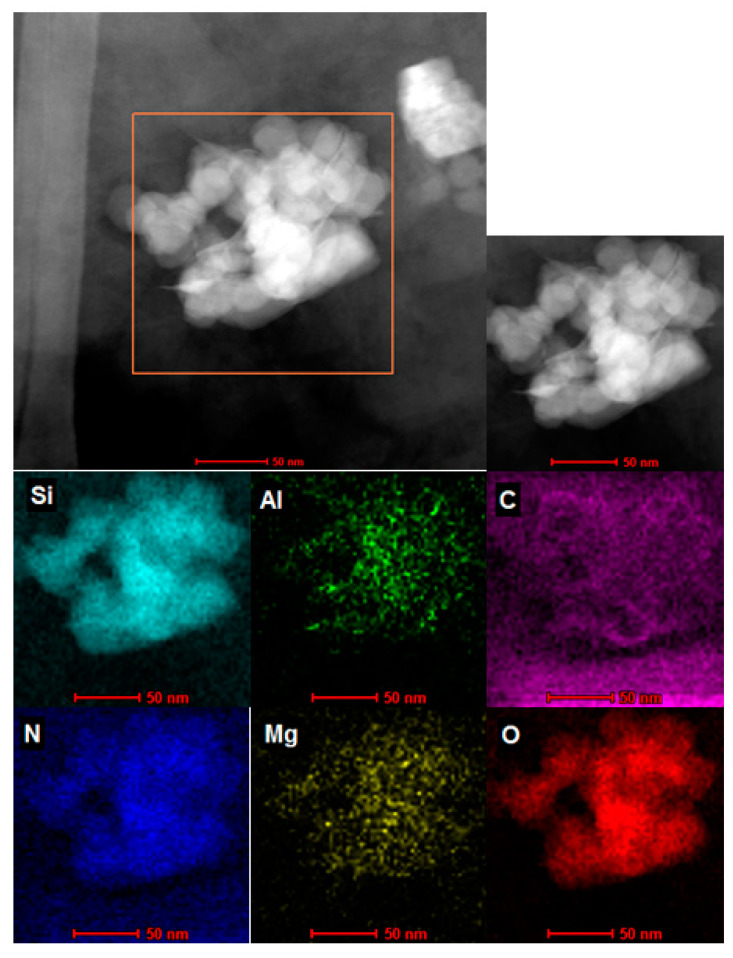
STEM EDS analysis of the N-SM/PAA-SS/CTAB sample. Top: STEM image of the analyzed object; bottom: chemical mapping showing the distribution of the atoms within the boxed area.

**Figure A7:**
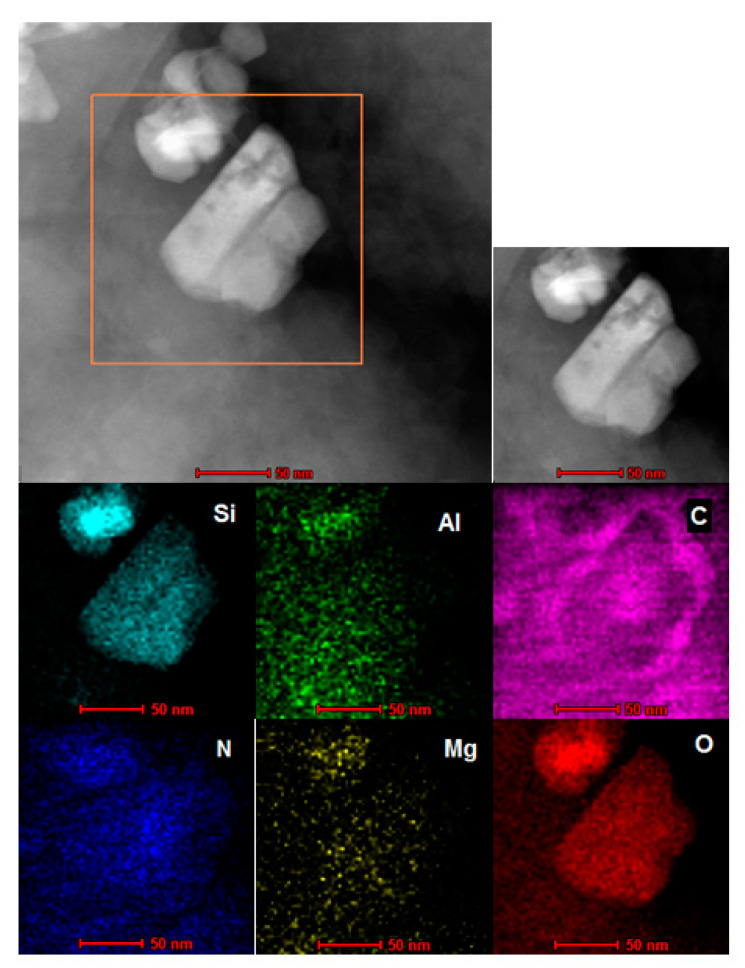
STEM-EDS analysis of the N-SM/PAA-HMW sample. Top: STEM image of the analyzed object; bottom: chemical mapping showing the distribution of the atoms within the boxed area.

**Figure A8:**
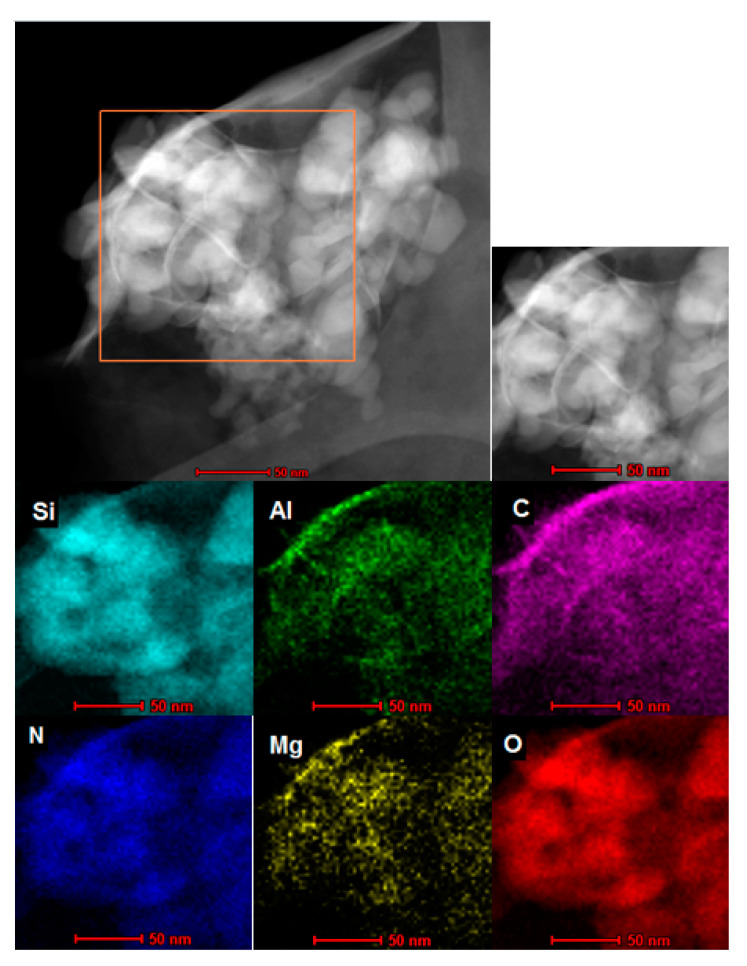
STEM-EDS analysis of the N-SM/PAA-HMW/CTAB sample. Top: STEM image of the analyzed object; bottom: chemical mapping showing the distribution of the atoms within the boxed area.

Figure A2, Figure A3, Figure A4, Figure A5, Figure A6, Figure A7 and Figure A8 present the STEM-EDS analysis of the studied samples. (Top: STEM image of the analyzed object; bottom: chemical mapping showing the distribution of the atoms within the boxed area). The STEM images of the N-SM, N-SM/PAA-SS, N-SM/PAA-SS/CTAB and N-SM/PAA-HMW/CTAB samples exhibit the spherical morphology of the lamellas. In contrast, the other samples (N-SM/PAA-LMW, N-SM/PAA-HMW, N-SM/PAA-LMW/CTAB) show the plate-like morphology. The distribution of the selected elements such as: Si, Al, C, N, Mg and O in the samples can be seen in the EDS maps. It can be observed that with the increase in the amount of polymer modifiers, the amount of carbon on the N-SM also increases. In the case of the N-SM/PAA-SS, N-SM/PAA-SS/CTAB, N-SM/PAA-HMW and N-SM/PAA-HMW/CTAB samples, the STEM-EDS analysis shows that the polymer is located at the lamellas edges and it accumulates on the contact surface of the lamellas.

### 2.4. IR Spectroscopy

**Figure 6:**
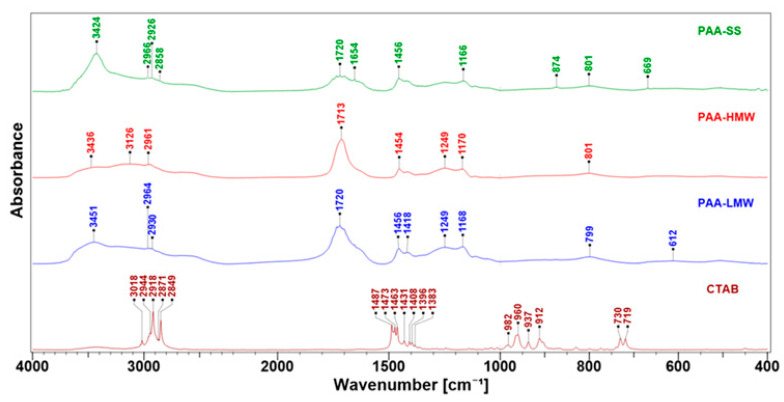
FT-IR spectra of the compounds used as stabilizers of the aqueous suspensions of the organoclay N-SM. (**Top**): star-shaped (PAA-SS), high molecular (PAA-HMW) and low molecular (PAA-LMW) polyacrylic acid; (**Bottom**)—hexadecyltrimethylammonium bromide CTAB.

FTIR spectroscopy was chosen to study the interactions between PAA, CTAB and N-SM organoclay. FTIR helps to confirm the adsorption process and gives additional information about the changes in the structure of used substances. The spectra of CTAB and PAA are presented in Figure 6. All PAA spectra showed –OH broad bands in the wavenumber range of 3700–2400 cm^−1^ due to the presence of adsorbed water (3451–3424 cm^−1^) and the intermolecular hydrogen bonds among the carboxyl groups, typical for hydrated PAA [53]. The weak bands in the range 2966 cm^−1^–2858 cm^−1^ were assigned to the ν(C–H) stretching vibrations in the polymer chain. The strong asymmetric band at 1720 cm^−1^ with the shoulder at 1654 cm^−1^ was classified as a superposition of the ν(C=O) stretching band of the carboxylic group and the δ(H–O–H) bending mode of water, the shape and amplitude of which depend on the contribution of both components. The absorption bands characteristic for the long carbon chains at 1456 cm^−1^ and 1418 cm^−1^ were also observed (scissors and bending vibrations of the CH_2_ and CHCO groups). The bands centred at 1249 cm^−1^ and near 1170 cm^−1^ are assigned to the δ(O–H) bending and ν(C–O) stretching vibrations of the neighbouring carboxyl group, respectively. In the low wavenumber range (below 1000 cm^−1^), the presence of bands with the maximum at 874 cm^−1^ and 800 cm^−1^ was associated with the occurrence of the γCH_2_ and γ(O–H) wagging vibrations from the carboxyl groups, respectively. The presented data are in agreement with other works [54,55,56,57,58]. The assignments of the main bands of CTAB are placed in Table 1.

**Figure 7:**
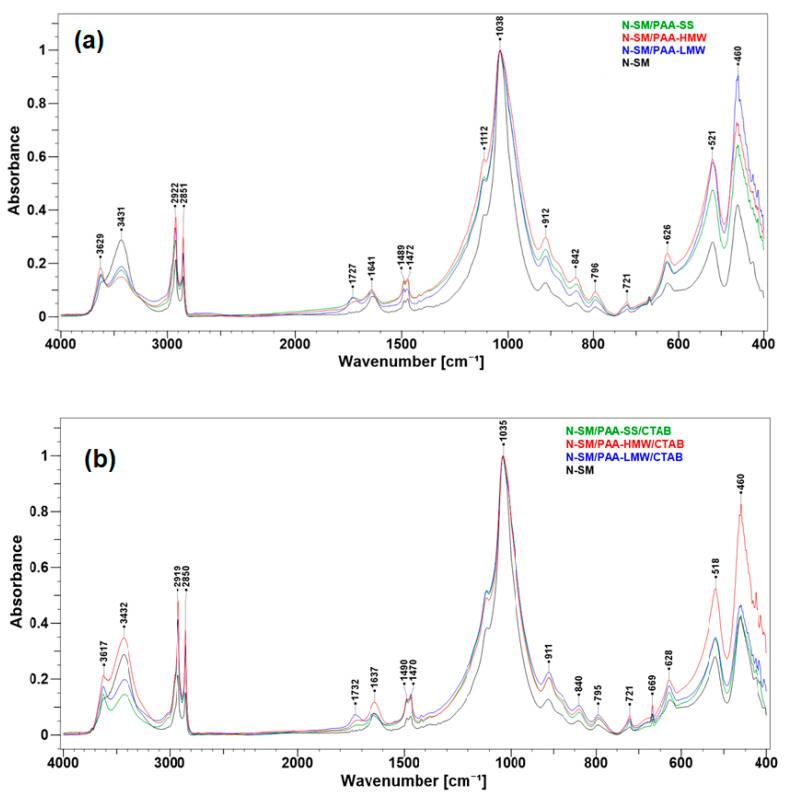
(**a**) FT-IR spectra of the neat organoclay N-SM and PAA-based composites: N-SM /PAA-LMW, N-SM/PAA-HMW and N-SM/PAA-SS; (**b**) FT-IR spectra of the organoclay N-SM compared to the PAA/CTAB composite materials: N-SM/PAA-LMW/CTAB, N-SM/PAA-HMW/CTAB and N-SM/PAA-SS/CTAB. All spectra were normalized on the band at 1038 cm^−1^ (**a**) and 1035 cm^−1^ (**b**).

The adsorption of PAA and CTAB on the surfaces of the N-SM was studied by FT-IR spectroscopy. The FT-IR spectra of all composites are demonstrated in Figure 7a,b. Particular attention was paid to the wavenumber range of 3700–2400 cm^−1^ and 1800–1400 cm^−1^, suggesting possible polymer and surfactant interactions with the support surface. As can be seen from the comparison, the FT-IR spectra of all modified nanoclays generally retain the profile of the host N-SM material. These spectra also showed that some absorption bands of both analytes in the wavenumbers of 1300–900 cm^−1^ are invisible due to overlapping with the very intensive bands of the untreated N-SM. The absorption bands at 3617 cm^−1^ and 3629 cm^−1^ are due to stretching vibrations of the structural –OH groups of montmorillonite in the nanoclay. The bands at 3430 cm^−1^ and 1640 cm^−1^ are attributed to the H–O–H stretching and bending vibrational modes, respectively. However, the partial desorption of water from the mineral surface after the PAA adsorption was observed. It was founded from the reduction in the corresponding bands’ intensity (Figure 7a). The same applies to the PAA/CTAB composites, except for the PAA-HMW/CTAB system, where the increase in the band intensity is observed (Figure 7b). A few broad bands appeared at 3451–2855 cm^−1^ in the solid PAAs spectra (Figure 6), which are related to the intermolecular hydrogen bonding within −COOH carboxylic group [53]. These signals disappeared in the spectrum of the modified organoclays (Figure 7a,b). Instead, a single asymmetric band centred at ~3430 cm^−1^ is displayed. Compared to the N-SM, the modified clays are characterized by the intense in the wavenumber range of 2922–2850 cm^−1^, which is assigned to the C−H stretching vibration mode in PAA macromolecules and the aliphatic chain of the surfactant molecules.

The FT-IR spectra of the nanocomposites reveal a new weak absorption band at ~1730 cm^−1^, which is assigned to the stretching vibrations of the carbonyl group C=O of the adsorbed PAA. The corresponding band of the neat polyacrylic acid appears in the 1720–1713 cm^−1^ region (Figure 6). The shift towards higher wavenumbers proved that regardless of the PAA molecular weight, the C=O groups are weaker hydrogen-bonded to the clay surface than in the solid PAA. These clearly confirm the PAA adsorption on the adsorbent surfaces.

The characteristic bands present in the CTAB spectrum (Table 1) are also preserved in the FTIR spectra of the composites (Figure 7b). The maintenance of the bands, relative to the symmetric and asymmetric stretching vibrations of CH_2_ in CTAB chain at 2919 cm^−1^ and 2850 cm^−1^ as well as the scissoring of N^+^(CH_3_)_3_ moiety between 1490–1470 cm^−1^ indicates that the hydrophobic tails groups do not interact directly with the adsorbent surface. The alkyl chains appear to interact with themselves to form the organic-rich layer on the organoclay surface that does not restrict the stretching vibration modes. Low intensities in the latter bands of the N-SM spectrum proves that the TMSA chains are mainly present between the montmorillonite layers attenuating the IR signal. The position of these bands does not change after PAA and PAA/CTAB adsorption; however, a slight increase in the intensity of these bands is observed (Figure 7a,b), which confirms the interaction of these compounds with the organoclay surface.

### 2.5. XRD Analysis

**Figure 8:**
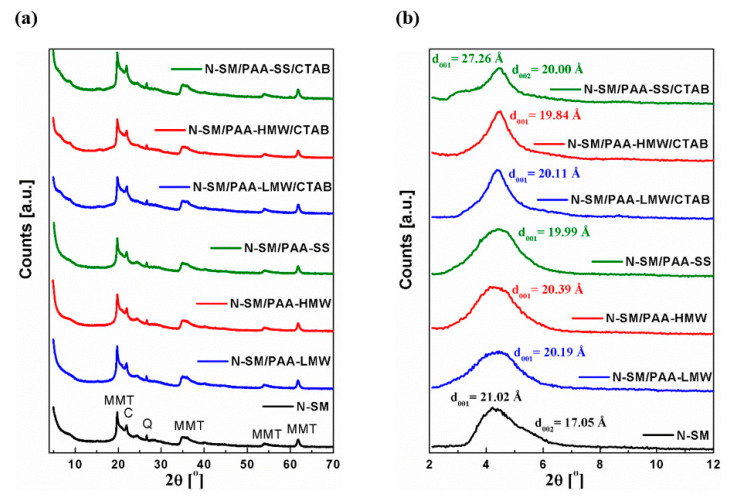
(**a**) X-Ray diffraction patterns of montmorillonite organoclay N-SM and N-SM modified by PAA and the PAA/CTAB mixture; (**b**) the corresponding SAXS diffraction patterns in the angle of 2°–12° 2θ (MMT = montmorillonite, C = cristobalite, Q = quartz).

**Figure A9:**
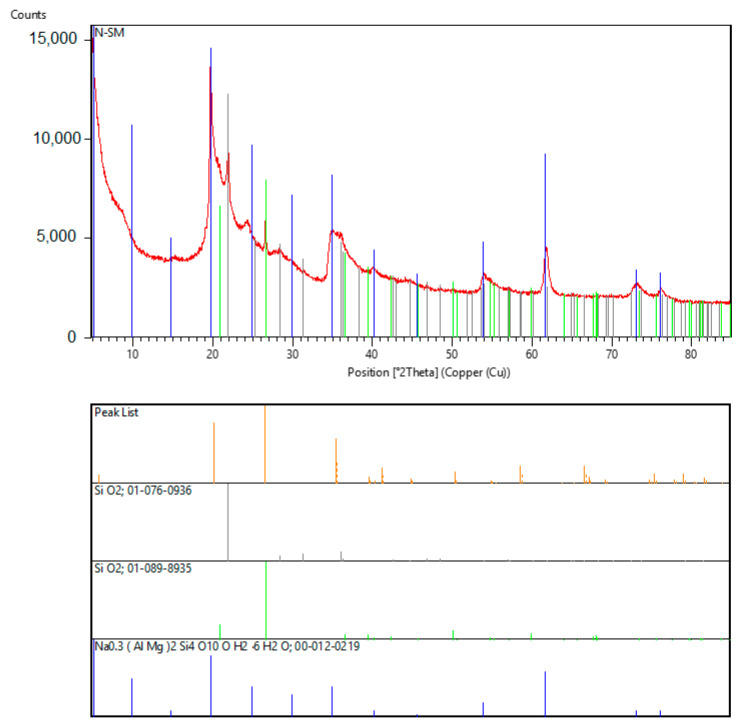
Identification of crystalline phases of the N-SM organoclay. The presence of montmorillonite, cristobalite and quartz was proved using the ICDD PDF4+2021 diffraction database.

**Figure A10:**
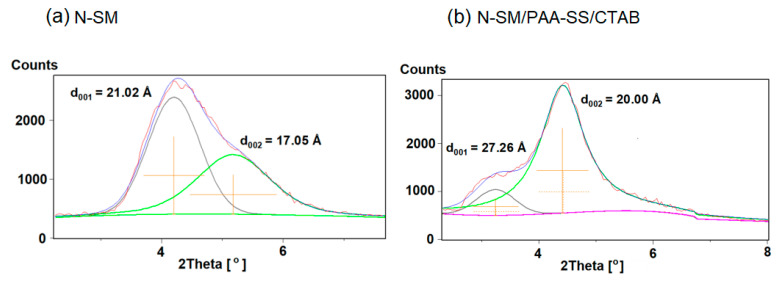
SAXS pattern of the basal diffraction peaks deconvoluted after fitting by the Gaussian function: (**a**) pure N-SM organoclay; (**b**) the N-SM /PAA-SS/CTAB composite.

X-ray powder diffractometry was used to evaluate the degree of interactions between the N-SM organoclay and that treated with PAA and/or the PAA/CTAB mixture. This method helps to analyze the structure of the adsorption layer, intercalation process and the ways of the adsorbent-adsorbate bonding, which is of great importance in the successful preparation of stable composites. Figure 8 shows the XRD patterns for all samples of the investigated clays in wide-angle scanning from 5° to 70° (Figure 8a) as well as small-angle 2°–12° 2θ scattering (Figure 8b). The basal spacing of the clay was derived from the peak position (d_001_ reflection) in the XRD patterns according to the Bragg equation (λ = 2dsinθ). The XRD pattern shows the N-SM organoclay to be composed mainly of montmorillonite whose characteristic reflections are positioned at 2θ = 19.75°, 34.98°, 53.84° and 61.69°. In addition, trace impurities of SiO_2_ also exist, and their assignments were as follows: 2θ = 21.88° (cristobalite) and 2θ = 26.61 degrees (quartz) (Figure 8a and Figure A9). The curves in Figure 8a show no changes in the diffraction reflexes of all organically modified samples, compared to the unmodified N-SM clay. Moreover, the SAXS analysis (Figure 8b) shows that besides the N-SM/PAA-SS/CTAB composite, the other types of PAA in the presence of CTAB molecules do not cause any significant changes in the gallery d-spacing, but rather affect the stacking of the silicate layers and the distribution of the basal spacings in the organoclays. The total difference in the basal spacings (Δd_001_) between the untreated and modified clay does not exceed 1Å. In the case of the N-SM/PAA-SS/CTAB sample, the increase in basal spacing d_001_ from 2θ = 4.20° (21.02 Å) to 2θ = 3.24° (27.26 Å) is observed probably due to the formation of a secondary layered structure with some galleries intercalated by the PAA-SS/CTAB complex (Figure A10). Presumably, in this system, a new structure is created between that narrow (17.05 Å) N-SM gallery. It now contains a layer composed of the primary TMSA chains together with the PAA-SS macromolecules and the CTAB molecules, which additionally increases the gallery d-spacing. Generally, the adsorbed PAA macromolecules and the PAA/CTAB complexes can interact with the surface groups of the clay particles rather than with those situated in the interlayer space [65]. The asymmetric basal spacing peak of the pure N-SM clay actually consists of two unresolved peaks, as evidenced from Figure 8b and Figure A9. This suggests the existence of a disorder in the stacking of silicate layers in the organoclay. The first d_001_ at 2θ = 4.20° (21.02 Å) is attributed to the arrangement of TMSA alkyl chains in the form of the paraffin-type monolayer tilted to the silicate layer [66,67,68,69,70]. The second peak d_002_ at 2θ = 5.18° (17.05 Å) evidences the gap size close to the flat bilayer of the alkyl chains of TMSA [64,65,71]. The presence of these two peaks shows the decrease in the basal spacing of some galleries with smaller packing densities [71]. The interlayer gallery spacing for the N-SM/PAA composites is independent of the PAA molecular type, as evidenced from Figure 8b. All of the obtained hybrid materials exhibit clear regular layered structures, demonstrated by the presence of a very similar distribution of d_001_ spacing and single diffraction peaks compared to the unmodified clay. The analogous situation is observed for the structures involving PAA/CTAB complexes. However, in the case of the N-SM/LMW-PAA/CTAB, N-SM/HMW-PAA/CTAB and the N-SM/PAA-SS/CTAB systems, there can be observed significant narrowing of the basal diffraction peaks, which are more evenly distributed. This indicates a more regular arrangement of layers than those present in the systems without CTAB. This proves that the PAA polymer and the CTAB surfactant adsorbed on the external clay surface influence the primary distribution of the layered sheets but keep the same d-spacing. Recently the PAA adsorption mechanism on bentonite has been described in detail [55]. According to the proposed model, the interactions of montmorillonite with the polyacrylic acid chains are based on the surface and edge adsorption, combined with the intercalation of the galleries between the silicate sheets.

However, in this study, the situation is different as we deal with an organically modified material where the interlayers are already occupied by the TMSA molecules. The organic treatment of the clay makes the normally hydrophilic montmorillonite hydrophobic, thus allowing it to interface with many different polymer matrices. It should be noted that for the organoclay used in this study, both the galleries and the outer surface of the N-SM particles are already covered with TMSA polyelectrolyte. As a result, the overall surface of N-SM clay remains positive. However, structural defects at layer edges give rise to additional PAA and a small amount of anion exchange capacity, too [72,73]. Given the anionic nature of the polyacrylic acid chains in the reaction medium, their electrostatic interactions with the positively charged head of the surfactant appear to be the most preferable [74]. Thus, the adsorption of the PAA molecules can take place both at the edges and on the external surface of the N-SM organoclays. However, in the PAA/CTAB system, the obtained polymer/surfactant complexes are probably adsorbed on the external surface of the N-SM particles. The structural analysis revealed no chemical bonding of the polymer chains to the montmorillonite surface regardless of the type of organic modification. Thus, the interactions between the components of the analyzed system are mainly of a physical adsorption and/or electrostatic nature. The XRD structural studies confirmed that there is no intercalation of the PAA molecules and the linear PAA/CTAB complexes into the montmorillonite interlayers. However, in the presence of CTAB molecules, the interactions between the long hydrophobic parts of TMSA and the CTAB chains will be preferred.

### 2.6. Zeta Potential Measurements

**Figure 9:**
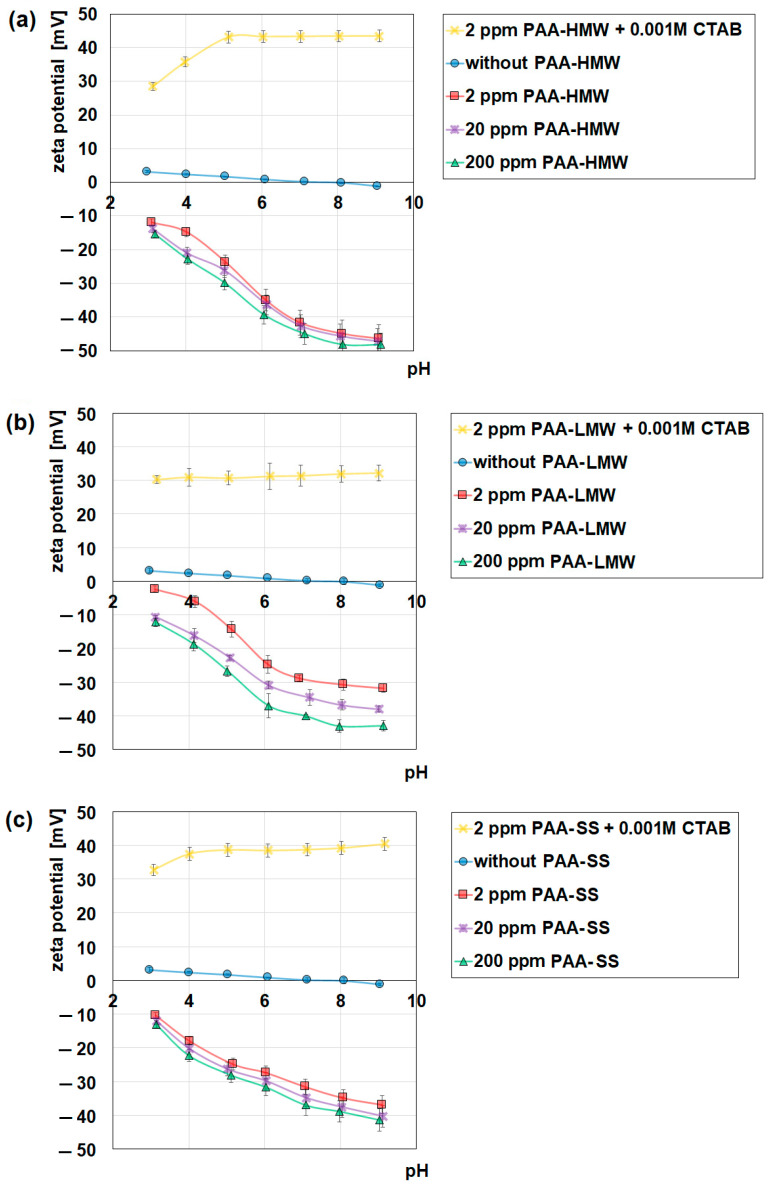
Influence of polyacrylic acid on the zeta potential of nanoclay, (**a**) PAA-HMW, (**b**) PAA-LMW, (**c**) PAA-SS.

The zeta potential measurements were used for the estimation of the electrokinetic potential of the studied systems, which is important in the characterization of the properties of the composites. Moreover, the zeta potential measurements help to estimate the stability of the studied systems using the obtained values of the zeta potential (systems are stable when zeta potential is higher than +30 mV or lower than −30 mV). Figure 9 shows the effects of the presence of polyacrylic acid and CTAB (0.001 mol/dm^3^) on the zeta potential (ζ) of the aqueous suspensions of nanoclays. The first observation is that the zeta potential values of the aqueous suspensions of nanoclay, with the increase in pH, do not change significantly in response to the pH changes. The isoelectric point of the nanoclay equals about 7.5–8, which means that up to this value, the surface of the studied adsorbent is positively charged and starts to become negative above it. Such behaviour is typical of the mineral clays [51]. The situation changes after the addition of polyacrylic acid. The presence of this polymer causes a significant decrease in the zeta potential. The observed decrease is the consequence of two effects: the shift of the slipping plane coming from the polymer adsorption towards the bulk of solution and the existence of the negatively charged groups in the diffused part of the electrical double layer. The above-mentioned effect is more noticeable when the larger concentration of the polymer is used, so as the concentration of PAA increases, the values of ζ decrease in each system. As far as the influence of the molecular weight of the polymer is concerned, it can be seen that if the molecular weight of PAA increases, the values of the zeta potential decrease (Figure 9a,b), which is the effect of the larger shift of the slipping plane coming from the adsorption of higher molecular weight PAA as well as a larger number of the negatively charged groups in the diffused part of the electrical double layer. Another observation from the obtained data concerns the influence of the polymer structure on the zeta potential. It can be seen that the presence of PAA-SS on the nanoclay surface influences the course of the zeta potential versus the pH curve in a different way than PAA-LMW with a comparable molecular weight value (Figure 9b,c). Moreover, in the case of PAA-LMW, the polymer concentration has a greater effect than in the presence of PAA-SS. This fact is a result of greater conformational flexibility of the linear polymer that can form, depending on the concentration and interactions with the surface, both flat, brush and coil conformations. In the case of the star polymers, the conformational flexibility is much smaller, and this fact translates into a noticeably smaller impact of the polymer concentration on the zeta potential in the studied systems. Moreover, as one can see, the best stability among the studied systems was obtained in the PAA/CTAB/N-SM systems where the values of the zeta potential were higher than +30 mV almost in all measured pH ranges. In the case of the systems without CTAB, their stability increases with the increase in pH, as these systems can be regarded as stable when pH is higher than six.

The addition of the cationic surfactant to the systems significantly changes the situation as the zeta potential increases in each measured system. The zeta potential has positive values of about 43 mV (PAA-HMW), 31 mV (PAA-LWM) and 38 mV (PAA-SS). The reason for that is the formation of the positively charged polymer-surfactant complexes (Figure A1) and their adsorption on the nanoclay surface. The charge coming from these complexes causes the increase in the zeta potential. Such high values of ζ also indicate higher stability of the system because a system can be considered stable if the zeta potential value is higher than 30 mV or lower than −30 mV [75]. It should also be emphasized that the influence of the presence of a positive charge in the diffusion layer on the zeta potential is so large that despite the shift of the slipping plane definitely occurring in the system, the increase in the zeta potential is observed.

### 2.7. Stability Measurements

**Figure 10:**
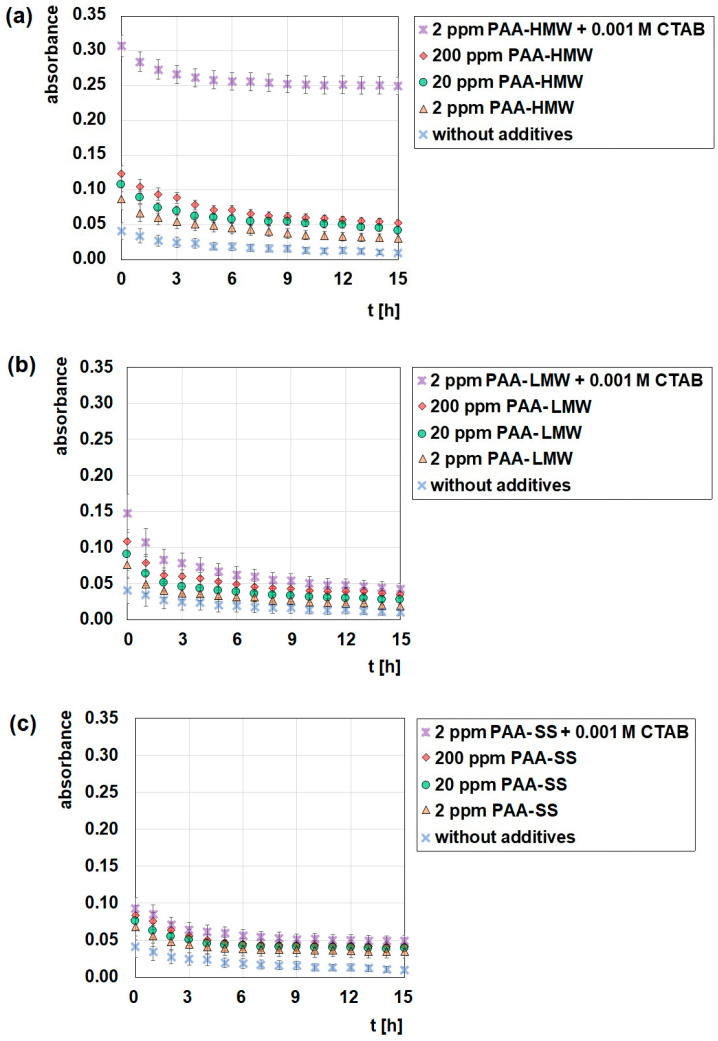
Influence of polyacrylic acid with the addition of CTAB (0.001 mol/dm^3^) on stability of the aqueous suspensions of nanoclay, (**a**) PAA-HMW, (**b**) PAA-LMW, (**c**) PAA-SS.

The UV-Vis spectrophotometry gives the answer to the question under which conditions the studied systems are the most stable and which of the factors has the greatest impact on stability. Figure 10 shows the influence of the presence and concentration of polyacrylic acid as well as the presence of CTAB on the stability of the aqueous nanoclay suspensions. It can be concluded that the aqueous suspensions of nanoclay without the addition of the polymer are completely unstable, as evidenced by the small absorbance values and their rapid decrease in time. The addition of polyacrylic acid to the systems increases stability in each measured system. This observation confirms that PAA can be used as an effective stabilizer of the aqueous suspensions of nanoclay. Because of the fact that regardless of its molecular weight and structure, PAA can adsorb on the surface of nanoclay, the most probable mechanism of stabilization is the electrosteric one [44]. The obtained data also show that the larger concentration of PAA, the more stable the systems are. The reason for that is a larger amount of the adsorbed polymer, causing more effective stability. Moreover, as the molecular weight of PAA increases, stability also increases slightly as a result of larger adsorption (Figure 1). As one can see, the star-shaped polyacrylic acid has the smallest influence on the stability of the studied systems, which is the consequence of its smaller adsorption as well as the smallest conformational flexibility.

The influence of surfactants on the stability and adsorptive properties of nanoclays is well known [76,77]. However, the above-mentioned studies concerned the usage of the organo-modified nanoclays synthesized with surfactants as adsorbents. In this paper, stability studies focus on the influence of CTAB, or more correctly, CTAB/PAA complexes, on the stability of the water suspensions of N-SM. An important observation from the obtained data is that the addition of cationic CTAB causes a significant increase in stability in all analyzed cases, but the most spectacular effect can be noticed in the PAA-HMW/CTAB/N-SM system. In the mentioned system, CTAB increases stability three times compared to the sample without the addition of surfactant. Interestingly, this absorbance value is maintained for 15 h, which indicates the best stability that has been achieved. The reason for this is the formation of the polymer-surfactant complexes, which are reluctant to adsorb at the nanoclay surface, but they are present between the nanoclay particles. The surface tension measurements show clearly that the polymer-surfactant complexes are formed in each of the tested systems, especially in the PAA-HMW/CTAB one (Figure 2), but also in each system, a decrease in the PAA adsorption in the presence of CTAB is observed (Figure 1). Therefore, the most probable mechanism responsible for stability in the systems with the surfactant is the depletion stabilization combined with the electrosteric stabilization [78]. The effective formation of complexes between PAA and CTAB remaining in the bulk of the solution and not adsorbing on the N-SM surface is responsible for the spectacular increase in the stability of the system. As has been shown, the addition of pure PAA-HMW to the suspension does not provide such good results. Therefore, in order to ensure the long-term stability of the aqueous N-SM suspensions, the usage of the PAA-HMW/CTAB complexes is the best solution.

## 3. Materials and Methods

### 3.1. Materials

Nanoclay was used as the adsorbent (montmorillonite clay contains 25–30 wt.% of trimethyl stearyl ammonium (TMSA)) purchased from Sigma-Aldrich ((Merck Group)—Darmstadt, Germany). The specific surface area of 11.16 m^2^/g, the pore volume of 0.09 cm^3^/g and the mean pore diameter of 30.9 nm were determined by the BET method (ASAP 2405 analyzer, Micromeritics (Norcross, GA, USA). The particle size distribution was also determined (d0.1 = 4.563 µm; d0.5 = 16.957 µm; d0.9 = 32.476 µm) using a laser diffraction particle size analyzer (Mastersizer 2000, Malvern Instruments (Malvern, United Kingdom)). The results of the adsorbent elemental analysis (EuroEA3000 CHNS-O Analyser, EuroVector (Pavia PV, Italy)) are as follows: N% = 1.312, C% = 22.302, H% = 4.401. In order to ensure the largest possible surface area and to remove possible contamination, the nanoclay was washed with the redistilled water until the conductivity of the filtrate was smaller than that of the redistilled water (about 2 μS/cm). The ED-XRF measurements confirmed the absence of impurities on the surface of the studied clay mineral.

**Figure A11:**
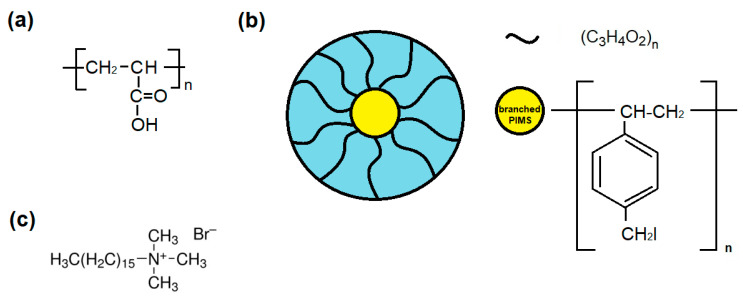
Structures of the compounds: linear PAA (**a**), star-shape PAA (**b**), CTAB (**c**).

Hexadecyltrimethylammonium bromide (CTAB) of the formula: [(C_16_H_33_)N(CH_3_)_3_]Br (Figure A11c) was purchased from Sigma-Aldrich. The results of the surfactant elemental analysis (EuroEA3000 CHNS-O Analyser, EuroVector (Pavia PV, Italy)) are as follows: N% = 3.992, C% = 63.045, H% = 11.923.

The other used chemical compounds were: NaCl (Sigma-Aldrich Merck Group)—Darmstadt, Germany)) used as a background electrolyte, benzethonium chloride (Hyamine^®^ 1622) (Sigma-Aldrich (Merck Group)—Darmstadt, Germany)) used to determine the amount of adsorption and HCl and NaOH solutions (Avantor Performance Materials Poland SA (Gliwice, Poland)) of various concentrations used for the pH adjustment.

Linear polyacrylic acids with the molecular weights of 100,000 Da (PAA-LMW) and 1,300,000 Da (PAA-HMW) (Figure A11a) were purchased from Sigma-Aldrich. The star-shaped polyacrylic acid (PAA-SS) with a molecular weight of 99,600 Da (Figure A11b) was synthesized for research purposes (all details are presented below). This polymer had 10 arms made of polyacrylic acid units and a core of poly[p-(iodomethyl) styrene] (PIMS). The polydispersity index (M_w_/M_n_) of this macromolecule equals 2.6. The results of the elemental analysis (EuroEA3000 CHNS-O Analyser, EuroVector (Pavia PV, Italy)) of the polymers are as follows: PAA-LMW: N% = -, C% = 46.667, H% = 5.4323, PAA-HMW: N%= -, C% = 47.913, H% = 5.376 and for PAA-SS: N% = 0.241, C% = 48.097, H% = 5.267. All polymer stock solutions were prepared by adding the appropriate amount of PAA to the redistilled water and then were placed on a magnetic stirrer for 30 min for the complete dissolution of the substance.

#### Synthesis of the Star Polymer with the Poly[p-(iodomethyl)styrene] Core and Poly(acrylic acid) Arms

The synthetic procedure towards the star polymer with a hyperbranched core and poly(acrylic acid) arms included two main steps: the synthesis of precursor star with the tert-butyl ester functionalities followed by their conversion to carboxylic groups. First, the precursor star polymer with the poly[(p-iodomethyl) styrene] core and the poly(tert-butyl acrylate) arms was synthesized using iodine mediated controlled radical polymerization, as described in [79], yielding the polymer of M_n_ = 175,000 Da. In the next step, the tert-butyl groups of the obtained polymer were hydrolyzed based on the procedure previously described in [80]. Briefly, the obtained polymer was dissolved in dichloromethane, and trifluoroacetic acid (five-fold molar excess of acid with respect to the tert-butyl groups) was added. After 24 h, the precipitated polymer was dissolved in water, and the same portion of trifluoroacetic acid was added to the reaction mixture. After the next 24 h, the reaction mixture was neutralized using NaOH and dialyzed to remove low molar mass products. Subsequently, water was evaporated, and the obtained product was lyophilized. In this way, 10-armed star polymer with the molar mass M_n_ = 99,600 Da was synthesized.

### 3.2. Methods

#### 3.2.1. Adsorption Measurements

In order to determine the amount of the PAA adsorption, the method described by Crummett and Hummel [81] was used. Before the adsorption measurements, the calibration curves were prepared. The PAA solutions at the concentrations: 20, 80, 100, 200, 300, 400, 600, 700, 800 and 900 ppm with a background electrolyte (0.01 mol/dm^3^ NaCl) in each sample were prepared. A 5 cm^3^ sample of these solutions were taken for further analysis and placed in the 25 cm^3^ flasks. 1 cm^3^ of a 20% NaOH solution was added to these flasks. They were sealed with a stopper and placed in a heated oven (90 °C) for 40 min. After this time, the samples were cooled to room temperature, and pH was adjusted to 10 in each sample. Then 1 cm^3^ of 1% hyamine solution (*w*/*w*%) was added to each flask, and the flasks were filled to the mark. The spectrophotometric measurements were made after 15 min at a wavelength of 500 nm (Cary 100, Varian Instruments (Palo Alto, CA, USA)). Each measurement was repeated four times.

After determining the calibration curves, the measurements of the polyacrylic acid adsorption in the absence and presence of 0.001 mol/dm^3^ CTAB on the nanoclay surface modified with the trimethyl stearyl ammonium groups were made. The solutions were prepared as those for the calibration curves, but this time 0.01 g nanoclay was added to each system. The obtained suspensions were shaken for 24 h. Then the adsorbent was centrifuged twice for 15 min. The amount of adsorption was determined spectrophotometrically (Cary 100, Varian Instruments (Palo Alto, CA, USA)) in an analogous manner to the method presented for the calibration curves. Each measurement was repeated four times, and the average values were reported.

#### 3.2.2. Surface Tension Measurements

The surface tension was determined based on the tensiometric measurements using the pendant drop method. The measurements were made using a Theta optical tensiometer (KSV Instruments Ltd. (Helsinki, Finland)). Fifteen flasks with a volume of 50 cm^3^ each were prepared for this purpose. The appropriate volume of CTAB was added into each flask to obtain the CTAB concentration of s in the range: 0.0001–0.01 mol/dm^3^ in each flask. For the series of measurements performed in the presence of polyacrylic acid, the appropriate amount of PAA was added to each flask so that its concentration equaled 100 ppm, the concentrations of CTAB were in the same range as previously. Each measurement was repeated five times, and the average values were reported.

#### 3.2.3. High Resolution Transmission Electron Microscopy (HRTEM)

The N-SM/PAA samples with and without CTAB, prepared in a manner analogous to those used during adsorption tests, were dried and ground to fine powders in an agate mortar. The obtained powder of each sample was poured with 99.8% ethanol (POCH) to form a slurry that was subsequently inserted into an ultrasonic homogenizer for 10 s (Omni Sonic Ruptor 400 (Omni International – Kennesaw, GA, USA)). Then the slurry containing samples were pipetted and supported on a 300 mesh copper grid covered with the lacey formvar and stabilized with carbon (Ted Pella Company (Redding, CA, USA)) and left on a filter paper for ethanol evaporation. The samples deposited on the grid were inserted into a single-tilt holder and moved to the electron microscope. The high-resolution electron microscope Titan G2 60–300 kV (FEI Company (Hillsboro, OR, USA)) was used to display the studied materials. Microscopic studies were carried out at an accelerating voltage of the electron beam equal to 300 kV for the materials.

The elemental mapping was made in the STEM-EDS mode by collecting the EDS spectrum of each of the corresponding pixels in the map point by point. The collected maps were then presented in the form of a matrix of coloured pixels with the intensity corresponding to the amount of the element.

Phase separation was performed with the FFT generated from the HRTEM images of the samples. Qualitative and quantitative contents of main elements in the studied materials were determined from the EDS spectra collected using the FEI Quanta 3D FEG scanning electron microscope, equipped with the EDS spectrometer.

#### 3.2.4. FT-IR Spectroscopy

The FT-IR spectra were obtained in the transmission mode by the KBr pressed disc method (4 mg of sample per 200 mg of KBr) with the mid-infrared range of 4000–400 cm^−1^ at the resolution of 4 cm^−1^ using a Nicolet 8700 FT-IR spectrometer (Thermo Fisher Scientific (Waltham, MA, USA)) equipped with DLaTGS (KBr window) detector, EverGlo mid-IR source and mid-IR optimized Ge-on-KBr beamsplitter. The resident Omnic software was used to collect and process the spectra. For each spectrum, 64 scans were averaged. The normalization and baseline correction functions were applied.

#### 3.2.5. XRD Studies

The diffraction data were collected using an Empyrean diffractometer ( Malvern PANalytical (Malvern, United Kingdom)) with the PIXcel^3D^ detector using the monochromated Cu-Kα radiation (λ = 1.542 Å), in the 2θ range of 5–70° with a step size of 0.026° and counting time 221.34 s. Wide-angle scanning (WAXD) was made in 2θ = 2–12° and 55.28 s. Sideways divergence of either the incident or scattered beam was controlled using the Soller slits (0.04 rad). The resident Data Viewer and HighScore Plus software were applied to collect and process the diffractograms.

#### 3.2.6. Zeta Potential Measurements

Solutions with the same parameters as for the measurement of the surface tension were prepared in 50 cm^3^ flasks. They were tested with a Zetasizer NanoZS (Malvern Instruments (Malvern, United Kingdom)). Before each measurement, the cell was rinsed with the studied solution. The software converted the electrophoretic mobility of the studied sample into the zeta potential using the Smoluchowski equation. Each measurement was repeated six times, and the average values were reported.

The polyacrylic acid suspensions with concentrations of 2, 20 and 200 ppm were prepared in 500 cm^3^ beakers with a background electrolyte (0.01 mol/dm^3^). Then 0.05 g of the surface-modified nanoclay (N-SM) was weighed and added to the solutions. The obtained suspensions were sonicated (Sonics Vibra Cell ultrasonicator, Sonics & Materials, Inc. (Newtown, Connecticut, USA)) for 30 s. After ultrasonication, the solutions were split into seven small portions and pH was adjusted to 3–9. Then the electrophoretic mobility was measured and recalculated into the zeta potential using the Smoluchowski equation. In the systems containing PAA and CTAB a concentration of PAA was equal to 2 ppm, whereas the surfactant concentration was 0.001 mol/dm^3^. The zeta potential was estimated in the way described previously.

#### 3.2.7. Stability Measurements

The effect of polyacrylic acid concentration and the presence of 0.001 mol/dm^3^ CTAB on the nanoclay suspensions was investigated spectrophotometrically. The solutions of the 10 cm^3^ total volume were prepared, containing: 0.005 g of washed nanoclay, 1 cm^3^ of the background electrolyte NaCl (0.1 mol/dm^3^) and the appropriate volume of redistilled water. The suspensions were sonicated (Sonics Vibra Cell ultrasonicator, Sonics & Materials, Inc. (Newtown, Connecticut, USA)) for 10 s. Then the appropriate volume of polyacrylic acid was added to the systems in order to provide the concentrations of 2 ppm, 20 ppm and 200 ppm. The last component of the suspensions was CTAB. It was added in such an amount to ensure the 0.001 mol/dm^3^ concentration. The prepared solutions were placed in a measuring quartz cuvette in the spectrophotometer (Cary 300, Varian Instruments (Palo Alto, CA, USA)) and were scanned for 15 h. The obtained results were presented as the dependences between the changes in the absorbance in a function of time.

## 4. Conclusions

Polyacrylic acid can be used as a stabilizer for aqueous suspensions of the nanoclay. This subject is very important because such systems can be used in industry for water treatment and purification. The most likely stabilization mechanism is the electrosteric one resulting from the effective adsorption of anionic PAA on the positively charged nanoclay surface. The addition of CTAB causes a significant increase in stability in all cases, which is the effect of the polymer-surfactant complexes formation. At the same time, due to the very strong interactions between the two adsorbates, these complexes do not adsorb so effectively on the N-SM surface, which was confirmed by the surface tension and zeta potential studies. The zeta potential measurements also show that the polymer-surfactant complexes are positively charged. The largest stability is observed in the case of the PAA-HMW with the addition of CTAB. It is this system that can be recommended as the best solution for industrial use because a small concentration of CTAB ensures very high stability without the use of a large polymer concentration. The PAA polymers and PAA/CTAB complexes adsorbed, especially on the clay surface, influences the primary distribution of the layered sheets but keep the same basal d-spacing. However, in the case of the star-shaped polymer, the formation of the secondary layered structure with some galleries intercalated by the PAA-SS/CTAB complex is observed. The TEM-EDS studies show that the lamellas in the N-SM aggregates are not evenly covered with the TMSA modifier. The distances between the atoms in the N-SM lamella are 3.90 Å.

The N-SM, N-SM/PAA-SS, N-SM/PAA-SS/CTAB, and N-SM/PAA-HMW/CTAB samples show the spherical morphology of the rounded lamellas. However, the N-SM/PAA-LMW, N-SM/PAA/LMW/CTAB and N-SM/PAA-HMW samples show the plate-like morphology. In the N-SM/PAA-SS and N-SM/PAA-HMW/CTAB samples, some exfoliated plates occur. The adsorption of the PAA macromolecules and PAA/CTAB complexes take place mainly at the plate edges and on the contact space between the sheets. The obtained results give light to the successful preparation of the novel N-SM/PAA/CTAB composite dedicated to water purification. 

## Figures and Tables

**Table 1:** FTIR band assignment of the CTAB surfactant.

Assignment ^a^	Wavenumber [cm^−1^]
Symmetric stretching of C–H of N^+^(CH_3_)_3_ moiety	3018
Symmetric and asymmetric stretching of –CH_3_ and –CH_2_– in the chain	2944, 2918, 2871, 2849
Symmetric and asymmetric C–H scissoring of N^+^(CH_3_)_3_ moiety	1487,1473,1463
Symmetric and asymmetric C–H scissoring of –CH_2_– and –CH_3_	1431, 1408,1396, 1383
C–N^+^ stretching	982, 960, 937, 912
Rocking mode of the –CH_2_– in the chains for n > 4	730, 719

^a^ Based on [59,60,61,62,63,64].

## Data Availability

Data is contained within the article or Appendix A.
